# Round the Hospitals

**Published:** 1919-04-19

**Authors:** 


### ROUND THE HOSPITALS.

Canterbury is doomed to lose an inspiring personality in the person of Miss A. M. Messum, matron of the General Kent and Canterbury Hospital for twenty-eight continuous years. The last four and a half years of her term of office were mainly devoted to work for the wounded from the war who were admitted to the hospital at Canterbury. Trained at St. Thomas's Hospital in 1882, Miss Messum was appointed directly after to be sister-in-charge of the late Sir William MacCormac's men's surgical ward at St. Thomas's, where she remained nine years, leaving on November 9, 1891, to take up her work at Canterbury. In these twenty-eight years Miss Messum has seen many changes, mainly for the better, including the building of the nurses' home, the introduction of the

**ROUND THE HOSPITALS—(*continued*).**

system of three years' training, with the payment of probationers, the establishment of a Samaritan Fund in aid of the poorer patients, a well-worked Linen League, the addition of two ward balconies to the old building, and protection for the nursing staff by the erection of a covered way from the hospital to the home. In addition, a system of central heating will shortly be in working order, and there is hope of considerable reconstruction of the Kent and Canterbury Hospital in the near future. Miss Messum has from the first been a strong upholder of the Royal National Pension Fund for Nurses, a policy in which she has always considered to be one of the best investments a nurse can make. Like so many of our best, most influential, and able Nightingale nurses, Miss Messum has never been photographed, except in a group, and we are therefore deprived of the privilege of publishing her portrait with this notice. Miss Messum is, we understand, to vacate office in June next, and her retirement will constitute a serious loss to Canterbury and its hospital, as well as to many friends, fellow workers, and supporters who know, honour, and value, at their true worth, one of the most interesting and engaging of our great provincial matrons.

---

In her way Miss Messum is a character whom it is a pleasure to meet, especially if the visitor has the privilege of being taken round the hospital by its matron. She is full of interesting reminiscences, and has a keen appreciation of loyal service, the best of nursing methods, and all that pertains to the care, nursing, and recovery of the patients. We shall be surprised if all who know Miss Messum do not make it their privilege to visit the Canterbury Hospital and spend a little time with her before she leaves its service. Intelligent people who value the voluntary hospitals and take an interest in the methods and the system pursued by those who, like Miss Messum, have done so much to raise the standards of work, and to introduce progressive development and improvements, might gain much if they were to take occasion to visit Canterbury before Miss Messum retires from office. Our columns have often been lightened by contributions from her pen. What she writes often possesses, as they have done, a practical importance, with quaint touches here and there that have made them welcome and sometimes even inspiring. The good wishes of an army of fellow-workers and compeers will follow Miss Messum into private life, though her active spirit is not likely to allow her time and valuable experience to prove less fruitful in the future than they have done in the past. Her personality is one that will endure and bear fruit long after the day on which she surrenders her work at Canterbury.

---

At the Investiture held at Buckingham Palace on April 10, the King personally conferred the decoration of the Royal Red Cross on the following ladies: First class—Sister Catherine Forrest, late Q.A.I.M.N.S.; Matron Selina Bland, Civil N.S.; Sister Mildred Forres, Canadian A.N.S.; and Matron Beryl Campbell, Australian A.N.S. Second class—Assistant-Matron Mary Bickerdyke and Sister Mary S. Richardson, Q.A.I.M.N.S. (R.); Sister Elsie Cassidy, Civil Hospital Reserve; Sister Nellie Ewright, Canadian A.N.S.; Sister Grace Burns, Sister Beatrice Gibbon, Sister Jessie McDonald, and Sister Florence Young, Australian A.N.S.; Miss Hilda Gerrard, Mrs. Mabel Pickering, Miss Mary Prowse, and Miss Jane Turnbull, V.A.D. His Majesty conferred the Albert Medal on Sister Gertrude Carlin, T.F.N.S., in addition to the Royal Red Cross (second class), and on Staff Nurse Harriet Fraser, T.F.N.S., and Sister Gladys White, B.R.C.S. (with the Royal Red Cross, second class). It will be remembered that these three ladies distinguished themselves by exceptional coolness and courage on the occasion when a fire occurred last October in No. 36 Casualty Clearing Station at Rousbrügge, in Belgium. The sisters and staff nurse assisted in carrying the patients from the operating-room under circumstances of extreme danger to all concerned. The King also conferred the Military Medal on Sister Jane Trotter, Q.A.I.M.N.S. (R.), and Miss Lilian Forse, V.A.D. Queen Alexandra received all the above ladies at Marlborough House subsequent to the Investiture.

---

At a second Investiture on Saturday the King conferred the following Royal Red Cross decorations: *The Royal Red Cross and Bar*—Matron Maude Blakely, Q.A.I.M.N.S. *First Class*—Sister Cecily Thompson, Q.A.I.M.N.S., and Sister Margaret Alexander, Civil Hospital Reserve. *Second Class*—Sister Christina Brace, Sister Mary Cain, Sister Marion Dew, Sister Maura McBreen, Sister Charlotte Morris, Sister Ethel Spicer, Staff Nurse Wilhelmina Lee, and Staff Nurse Rosa Rothwell, all of Q.A.I.M.N.S.R.; Sister Janet Linton and Staff Nurse Nina White, Territorial Force Nursing Service; Matron Letitia Green and Sister Martha Young, of the Civil Nursing Service. The King also conferred the Military Medal on Miss Molley Bianconi, First-Aid Nursing Yeomanry, and Sister Eleanor Thompson, Canadian Army Nursing Service.

---

Queen Alexandra's visit to open the Edith Cavell Nurses' Home at the London Hospital on April 11 was tinged with sadness as the first public event at the institution since the death of Miss Luckes. Her Majesty was accompanied by Princess Victoria, Prince Valdemar of Denmark, and the Prince's daughter, Princess Margaret, and the Royal party was received at the door of the house by Lord Knutsford, Chairman of the Hospital, the new Matron, Miss Monk, and the Treasurer, Mr. W. T. Paulin. In the nurses' sitting-room the Danish Minister was in attendance with members of the Danish Committee in London who helped to collect £7,000 towards the Million Half-Crowns Fund. Lord Knutsford alluded to the fact that the Home was to have been called after Queen Alexandra, and that it had received the name of Edith Cavell by Her Majesty's express wish.

**ROUND THE HOSPITALS—(*continued*).**

Queen Alexandra, from a small platform directly in front of Sir George Frampton's bust of Edith Cavell, said: "I am delighted to open the Home in memory of our never-forgotten Miss Cavell and our dear Miss Luckes, who was everything to this Hospital." It was a very touching scene, and one which will not soon be forgotten. The linking of the great matron's name with that of her martyred pupil moved all present.

The King has sanctioned the promotion of Dame Emma Maud McCarthy to be Lady of Grace of the Order of the Hospital of St. John of Jerusalem in England.

A very long list of awards of the Royal Red Cross, of the Bar, and the first and second class of the Order was issued on April 9, and will be found in the *Times* of the 10th inst. The list comprises members of all the British nursing services, civil and military, as well as members of the Canadian Army Medical Corps, the Australian Army N.S.; the New Zealand A.N.S., and the South African N.S. The New Zealand Red Cross has been awarded to three members of Voluntary Aid Detachments.

The Central Council for District Nursing in London has adopted a resolution which reads:—"That it be a condition of a grant from the Council that the minimum commencing salary of a resident nurse shall be not less than £50 clear; and that the minimum in the case of non-resident nurses be £50 clear over and above a reasonable allowance paid in consideration of board and lodging." The Council itself has resolved that the salaries of district nurses should be increased.

A nurses' Registration Bill passed through the Standing Committee of the House of Commons on the 14th inst., and was ordered to be reported to the House. We strongly urge every matron, sister and nurse and the managers of the voluntary hospitals to read carefully the full report of the proceedings of the Standing Committee on the Bill, which will be found on page 69 *et seq.*

We note in the Annual Report of the General Hospital, Birmingham, that "the serious shortage of probationers (which is general throughout the country) gives cause for much anxiety. The number of the nursing staff has been, on an average, twenty below its proper level during the whole year." The facts point to a general levelling-up of the conditions of female labour in the district, and the management ought, we think, to examine closely into the reasons for this failure of the hospital to attract sufficient probationers. Are the hours too long? Is the pay inadequate? Would the institution of an eight hours' day or the offer of the popular one day's rest in seven alter the situation? In that case it is clearly in the best interests of the hospital that some such concession should be made. A nursing department run with but some five-sixths of its proper number is in a parlous state and demands immediate attention.

At St. George's Hospital influenza took heavy toll last autumn. The lately issued annual report states that no fewer than fifty-three members of the nursing staff were incapacitated at one time, and the utmost difficulty was experienced in carrying on. One of the nurses died. The out-patient department was wisely closed for a month at the height of the epidemic. An interesting new departure chronicled is the reception of nurses from children's hospital training-schools of not less than 100 beds as second-year probationers. This concession to children's hospitals has long been overdue, and we trust may command imitation.

After some hesitation it has been decided that a maternity hostel shall be at once established in Canterbury in connection with the Canterbury Maternity Association. It is proposed to rent the old Vicarage adjoining the new Health Centre for the city, in order to accommodate a few maternity patients—a plan which the nurses have long considered a necessary adjunct to their work. Strong support was given to the scheme by Mr. Stutchbury, of the Local Government Board, who said they could count on contributions of 50 per cent. from the Board for both initial and running expenses.

The principle of Mothers' Pensions was accepted by the Government in the House of Commons on April 8. Mr. Tyson Wilson moved "That in the opinion of this House, pensions adequate for a healthy and useful life should be paid to all widows with children or mothers whose family breadwinner has become incapacitated, such pensions to be provided by the State and administered by a Committee of the Municipal or County Council, wholly unconnected with the Poor-Law." Major Astor, while agreeing with the principle, asked that full consideration of the matter should be deferred till the Poor-Law is dealt with under the Ministry of Health Bill.

The Dollis Hill House Hospital has come to an end after a term of fine work for the wounded. The Bishop of Willesden took part in the ceremony of closing down, and gave his blessing to the little band of nurses. He made his hearers smile by his anecdote of a great surgeon who, using the Latin formula, told students round the bed of a patient that "operation is to be performed on this vile body." To his amaze there came from the bed the reply, in the same language, "Not so vile but that Christ was crucified for it."

The extent to which hospital sisters have hitherto been compelled to gamble for the future prospect of becoming matrons is illustrated by the letter of an assistant matron published in the *Morning Post.* She states that she received five years' training in two important London hospitals, and was fully qualified in all branches of nursing at the age of thirty. Her salary during these years of learning ranged from £10 to £20 a year. She was then promoted successively to the posts of ward sister, night superintendent, home sister,

**ROUND THE HOSPITALS—(*continued*).**

and assistant matron in different hospitals, two of which were training-schools for nurses. In none of these posts did she receive more than £36 a year, "the excuse being that the 'experience' was valuable as qualifying for the chief and highest post of matron." On joining the Army she was reduced to the status of a staff nurse for one year before being promoted sister at £50 a year. She is now nearing the age limit of forty-five. This is an absolutely true epitome of the career of hundreds, perhaps thousands, of educated, highly trained nurses, with wide administrative experience. Recent action taken by hospital authorities in London and the Provinces is striking at the root of this pernicious form of sweating.

---

It seems hardly credible that any V.A.D.s should be labouring under a sense of injury on account of not having been "thanked" for their services. But an "officer patient" in the *Manchester Despatch* declares they are being demobilised "with very little warning, and without a word of thanks." True to tradition, the Army style in demobilisation letters is curt to say the most. It is from the local authorities and the county organisations that the recognition is to come. We have had many instances brought to our notice of services, even the most exiguous, acknowledged beyond all expectations, and we can only advise those who believe themselves to be overlooked to wait and see.

---

Public attention has been irresistibly attracted to problems of nutrition during the rationing period, and it is not remarkable that learned societies have been holding conferences and issuing reports relating to the quantity and quality of the food best suited to maintain health and efficiency. The Report of the Food (War) Committee of the Royal Society, lately issued, modestly confesses that we know far too little about the science of nutrition to be dogmatic. One point elucidated is of value to hospital workers. It seems to be pretty clearly established that in order to carry on mental work, whether of high or low order, efficiently, it is essential that the processes of digestion should not make too great a call on the energies of the body. In other words, workers who have to put in some hours of close application during the afternoon should eat a light lunch and take their most substantial meal at 6.30 or 7, when work for the day is over. Thus the girl clerks who incur the disapproval of Dr. Howarth, the medical officer of health for the City of London, by taking a slight meal in the middle of the day are probably acting in self-defence. Unfortunately, they are not, we fear, always in a position to redeem the pledge to their digestions at a later hour. There is much to be said for the now popular custom of giving the sisters in hospitals late dinner.

---

Great efforts are being made under the Ministry of Reconstruction to redeem domestic service from unpopularity. The time is ripe for some *rapprochement* among institutions with a view to constituting a general service branch of hospital workers graded according to length of service and ability and provided with an attractive name and a neat uniform. The importance of the name cannot be overlooked, as may be seen from Government endeavours to get the word "chemist" rehabilitated. The word "domestic" is no longer approved. That of "servant" is entirely obsolete. Why not "hospital aids"? There is need for co-operation between institutions in order to make such a service really popular, for the grades ought to be permanent, so that a worker who has once attained to Grade I., for instance, could pass to another institution, should it be convenient to her, without loss of status.

---

A touching account of Miss Edith Cavell's last hours has been given by her friend, the Rev. H. T. S. Gahan, British Chaplain in Brussels. He explained the extraordinary recklessness of the men she succoured, some having even written postcards to her from London to give news of their escape, thus furnishing deadly evidence against her. During his last interview with her she told Mr. Gahan she was thankful for the quiet time in solitary confinement as her life had been such a rush and burden. She was not sorry to go; she was weary beyond endurance. "They have treated me very kindly here," she said. "I expected my sentence, and I am glad to die for my country. In the sight of Eternity I know now it is not enough to love your own. You must love all and not hate any." After the Communion service they said the hymn "Abide with me" softly together. Then she smiled and shook hands, saying "We shall meet again."

---

Miss Macmillan, who is a member of the London County Council, gave an interesting address on the value of outdoor nurseries for little children at a meeting of the Hornsey Branch of the National Council of Women. She expressed the hope that in time the elementary schools would be changed, some of the walls taken town, verandahs thrown out, baths and dining-room installed, and a new kind of training given to some of the teachers, who should work in shifts. She described the garden nursery at Deptford, recently visited by the Queen, where in the heart of one of the worst slums in London they turned out "magnificent, rosy, healthy, strong, intelligent children, with splendid teeth, fine vision, bodies as strong and supple as an antelope's, skin as smooth as a flower, and hair like silk."

---

The National League for Health, Maternity, and Child Welfare, which includes in its fold seven subsidiary societies, has issued its annual report for 1918. The main event of the year was the magnificent gift of £15,000 from the American Red Cross Society to be spent on establishing and maintaining for one year certain small maternity hostels or lying-in homes for poor mothers, with special provision for the Stepney School for Mothers and for the care of children of the professional classes whose mothers are bread-winners.

